# Pertussis in Soldiers, Israel

**DOI:** 10.3201/eid1103.040672

**Published:** 2005-03

**Authors:** Eyal Klement, Itamar Grotto, Itzhak Srugo, Naday Orr, Jacob Gilad, Dani Cohen

**Affiliations:** * Israel Defense Force, Medical Corps, Ramat-Gan, Israel;; † Tel-Aviv University, Tel-Aviv, Israel;; ‡ Bnai Zion Hospital, Haifa, Israel;; § Technion-Israel Institute of Technology, Haifa, Israel;; ¶ Soroka University Medical Center, Beer-Sheva, Israel

**To the Editor:** The role of adults as reservoirs of pertussis was previously well established (1–7). Young army recruits undergoing basic training in the Israeli Defense Force constitute a unique adult population because of their special living and service conditions. This and the fact that they are not vaccinated with the diphtheria, tetanus, and pertussis (DTP) vaccine after the age of 1 year (unlike children in most of the Western countries) led us to hypothesize that this semiclosed population may have an exceptionally high risk for pertussis. These young soldiers are on leave on weekends, during which time they come in close contact with susceptible family members, including young infants, and may thus facilitate the "import" and "export" of pertussis between the military setting and the general population. An outbreak of pertussis that recently occurred among infantry soldiers (8) indicated the need to conduct the present study, in which we sought to evaluate the prevalence and incidence of pertussis among young soldiers in the Israeli Defense Force.

We conducted 2 concurrent studies. The first was a 15-month (November 2001–March 2003), laboratory-based surveillance study of pertussis, which included 110 trainees who complained of persistent coughing (case definition: cough lasting 10–90 days) upon admission to compound clinics. Samples obtained from these patients were tested by a commercial enzyme-linked immunosorbent assay (ELISA) (PanBio, East Brisbane, Queensland, Australia) for the presence of immunoglobulin (Ig) A against a *Bordetella pertussis* sonicate and by an in-house IgA-detection ELISA directed against pertussis toxin as previously described (9). Results for IgA to *B. pertussis* sonicate were calculated as arbitrary ELISA units obtained according to the manufacturer's instructions. A result of 9 U (a cutoff point that was previously shown to provide a 98.5% specificity for the diagnosis of recent pertussis infection [8] or higher was considered positive. Results for IgA to pertussis toxin were calculated as arbitrary ELISA units according to a calibration curve of a serially, double-diluted, positive standard. The cutoff point was calculated by adding 3 standard deviations to the mean value of a group of 40 healthy study participants. A positive result in either test was considered a confirmed case of pertussis.

We conducted another substudy to estimate incidence. This substudy included 278 trainees who were interviewed regarding the occurrence of persistent cough and seeking of medical care during the preceding 6 months. We multiplied the prevalence of laboratory-confirmed pertussis found in the first study by the incidence of study participants with persistent cough who sought medical care in this study. The result was multiplied by 200,000 to receive incidence estimation for 100,000 person-years.

The median duration of cough among the 110 case-patients was 14 days, their median age was 19 years, 94 (85.5%, 95% confidence interval [CI] 77.5%–91.5%) were males, 71 (64.5%, 95% CI 54.9%–73.4%) were born in Israel, and 85 (77.3%, 95% CI 68.3%–84.7%) were in basic training when they visited the clinic. Twenty (18.2%, 95% CI 11.5%–26.7%) and 14 (12.7%, 95% CI 7.1%–20.4%) of the patients were positive for IgA antibodies to *B. pertussis* and pertussis toxin, respectively. Twenty-five patients (22.7%, 95% CI 15.3%–39.7%) were positive by either test. Significant variations were recorded during the follow-up period. The first period (November 2001–May 2002) was characterized by a high prevalence of pertussis among the 72 patients enrolled, with 19 (26.4%, 95% CI 16.7%–38.1%) and 14 (19.4%, 95% CI 11.1%–30.5%) positive for IgA to *B. pertussis* and pertussis toxin, respectively. In the second period (August 2002–March 2003), although characterized by the same median duration of cough (14 days), a substantially lower prevalence of pertussis was observed among the 38 patients enrolled, with only 1 (2.6%) and 0 patients, respectively, showing positive results in either of the 2 tests (p < 0.01 for differences between the 2 periods for both diagnostic methods).

The frequency of clinical symptoms observed in patients positive for pertussis by at least 1 ELISA (n = 25) was similar to those observed in patients negative for pertussis by both ELISAs (n = 85), with the exception of post-tussive emesis (40% versus 25%, respectively) and fever (4% versus 21%). These differences were not significant.

Of the 278 basic training respondents, 17 (6.1%, 95% CI 3.6–9.6%) reported a persistent cough (>2 weeks) during the preceding 6-month period; 13 (4.7%, 95% CI 2.5%–7.9%) had sought medical care. When we extrapolated from this sample and from the laboratory-confirmed prevalence of 22.7% among patients with persistent coughing who sought medical care (and thus came to the clinics), the incidence rate was 2,132 cases per 100,000 person-years (95% CI 440–6,240).

The prevalence of pertussis found in this study is comparable with that previously reported among U.S. Marine corps trainees, university students, and other civilian adult populations (1–7). Complete case-capturing could not be performed. However, the high clinical similarity between pertussis-positive and other cases of prolonged cough renders selection bias unlikely. The prevalence of disease in this study showed significant changes in relation to time in contrast to previous studies (3). This difference may be because our present study was conducted in a semiclosed population characterized by epidemic occurrence of the disease.

The incidence of pertussis reported in this study (2,132 cases per 100,000 person-years) is substantially higher than the findings of Nennig and others (10) among an urban population in San Francisco (176 cases per 100,000 person-years). This finding may be due to the difference in immunization practices between Israel and the United States (5 doses of DTP vaccine in the United States with the last 1 administered between the ages of 4 and 6 years, compared with only 4 doses of the vaccine during the first year of life in Israel) or to crowded living conditions of the recruits. Our findings emphasize the need for revaccination against pertussis of young adults in Israel, primarily of those at high risk for pertussis, such as army recruits.
